# Molecular Docking Study of Conformational Polymorph: Building Block of Crystal Chemistry

**DOI:** 10.1155/2013/309710

**Published:** 2013-10-23

**Authors:** Rashmi Dubey, Ashish Kumar Tewari, Ved Prakash Singh, Praveen Singh, Jawahar Singh Dangi, Carmen Puerta, Pedro Valerga, Rajni Kant

**Affiliations:** ^1^Department of Chemistry, Faculty of Science, Banaras Hindu University, Varanasi 221005, India; ^2^Institute of Pharmaceutical Sciences, Guru Ghasidas Vishwavidyalya (A Central University), Bilaspur, Chhattisgarh 495009, India; ^3^Departamento de Ciencia de los Materiales e Ingenieria Metalurgica, Facultad de Ciencias, Campus Universitario del Rio San Pedro, 11510 Puerto Real, Spain; ^4^Department of Physics, Jammu University, Jammu 180016, India

## Abstract

Two conformational polymorphs of novel 2-[2-(3-cyano-4,6-dimethyl-2-oxo-2*H*-pyridin-1-yl)-ethoxy]-4,6-dimethyl nicotinonitrile have been developed. The crystal structure of both polymorphs (**1a** and **1b**) seems to be stabilized by weak interactions. A difference was observed in the packing of both polymorphs. Polymorph **1b** has a better binding affinity with the cyclooxygenase (COX-2) receptor than the standard (Nimesulide).

## 1. Introduction

Polymorphism “Supramolecular isomerism” is pertinent to supramolecular chemistry, and crystal engineering in the same way as isomerization is pertinent to organic molecules. In the simplest way, polymorphism is the ability of molecules to produce more than one crystal structure [1, 2], resulted from interplay of kinetic and thermodynamic parameters [3]. The complexities of the organic solid state and especially the differences of intermolecular forces influence crystal packing [4]. Conformational polymorphism will always be a possibility for molecules that have multiple conformational isomers accessible energetically: every different conformation is a different molecular shape and can, in principle, form its own crystalline polymorph (or polymorphs) [5]. Because of the variation in crystallization environment (e.g., temperature, solvent, using of additives, and concentration), the same molecules can pack differently and form different crystal lattices or polymorphs [6–8]. As a result, the physical, chemical, and mechanical properties of the crystals can be dramatically affected. Nicotinonitrile-based crystals are highly influenced by *σ* and *π* cooperative effects [9]. Self-assemblies of these derivatives are governed by various weak interactions [10–20]. The presence of various weak interactions leads to the development of polymorphism in compounds [21–25]. Polymorphism in organic and inorganic solids can be of crucial importance in the drug design and pharmaceutical industries due to its regulatory action [26–28]. Earlier we had studied weak interactions and its polymorphism in 1,3-bis(4,6-dimethyl-1H-nicotinonitrile-1-yl)1,3-dioxy propane, which was symmetrical dimer [29]. This current study is focused on the pharmaceutical property of dissymmetrical molecule, 2-[2-(3-cyano-4,6-dimethyl-2-oxo-2*H*-pyridin-1-yl)-ethoxy]-4,6-dimethyl nicotinonitrile, and its polymorphs (**1a** and **1b**).

## 2. Experimental 

### 2.1. Synthesis of 2-[2-(3-Cyano-4,6-dimethyl-2-oxo-2H-pyridin-1-yl)-ethoxy]-4,6-dimethyl-nicotinonitrile

To a solution of 3-cyano-4, 6-dimethyl-2-oxo-nicotinonitrile (3 g, 0.02 mole) in 10 mL dry DMF, potassium carbonate (2.68 g, 0.02 mole) was added and the mixture was stirred for 2 h. 1,2-Dibromo ethane (0.02 mole) was added to it and stirred for 15 h. Completion of reaction was monitored through TLC. Solvent was removed on a rotary evaporator and residue was extracted in chloroform: water (1 : 1) (3 × 100 mL). Organic layer was dried with anhydrous sodium sulfate. Compounds were purified by column chromatography (50% EtOAc: hexane) leading to crude product as a yellow powder. 

*Yield*. 1.17 g (36%); ^1^H-NMR (CDCl_3_), *δ* 2.40 (s, 6H, CH_3_), *δ* 2.63 (s, 6H, CH_3_), *δ* 4.45 (t, 2H, *J* = 6, CH_2_), *δ* 4.72 (t, 2H, *J* = 6, CH_2_), *δ* 6.06 (s, 2H, ArCH), *δ* 6.69 (s, 2H, ArCH). ^13^C-NMR (CDCl_3_) *δ* 19.94 (CH_3_), *δ* 20.80 (CH_3_), *δ* 21.73 (CH_3_), *δ* 24.33 (CH_3_), *δ* 44.54 (NCH_2_), *δ* 64.37 (O*C*H_2_), *δ* 93.50 (CCN), 101.27 (CCN), *δ* 109.64 (CN), 115.04 (CN), *δ* 115.33 (Ar-CH), *δ* 118.08 (Ar-CH), *δ* 151.98 (CCH_3_), *δ* 154.37 (CCH_3_), 158.41 (CCH_3_), *δ* 160.93 (CO), *δ* 163.19 (COCH_2_). IR (KBr) cm^−1^: 659–848 (CH bending), 1156–1203 (COC, NC stretching), 1410–1595 (C=C stretching), 1650 (CO stretching), 2219 (CN stretching), 2858–2924 (CH, CH_3_, and ArH stretching). Elemental analysis for C_24_H_22_N_4_O_2_: Calcd. C; 62.42%, H; 5.20%, N; 16.18%, found: C; 62.40%, H; 5.19%, N; 16.19%; MS (FAB): *m*/*z*: 346 (*m* + 2).

### 2.2. Instrumentation

The X-ray diffraction measurements were carried out using a CrysAlis CCD, Oxford diffractometer. The structure was solved by direct methods with the SHELXS-97 program and refined by the full-matrix least squares method on *F* ^2^ data using the SHELXL-97 program. Molecular graphics: ORTEP; software used to prepare material for publication: MERCURY-3.1. FT-IR spectra were recorded on a VARIAN 3100 FT-IR spectrometer, which was evacuated to avoid water and CO_2_ absorptions, at a 2 cm^−1^ resolution in KBr. The 1H and 13C NMR spectra were recorded on a JEOL AL300 FTNMR spectrometer operating at 300.40 and 75.46 MHz for proton and carbon 13, respectively. The 1H and 13C chemical shifts were measured CDCl_3_ solution relative to TMS. The details of the data collection and final refinement parameters are listed in Table 1 and in the supplementary Material available online at http://dx.doi.org/10.1155/2013/309710.

## 3. Results and Discussion

Freshly synthesized 2-[2-(3-cyano-4,6-dimethyl-2-oxo-2H-pyridin-1-yl)-ethoxy]-4,6-dimethyl-nicotinonitrile was recrystallized in two different mixtures of solvent. Using mixture of Ethyl acetate-n-hexane (9 : 1) solvent, hexagonal crystals of pale pink color was obtained after 2 days at room temperature. However, recrystallization from a mixture of (1 : 1) chloroform-*n*-hexane was attempted, resulting in the appearance of light yellow, prismatic crystals (**1b**), at a temperature of −5°C (refrigerated).

**Figure 1:**
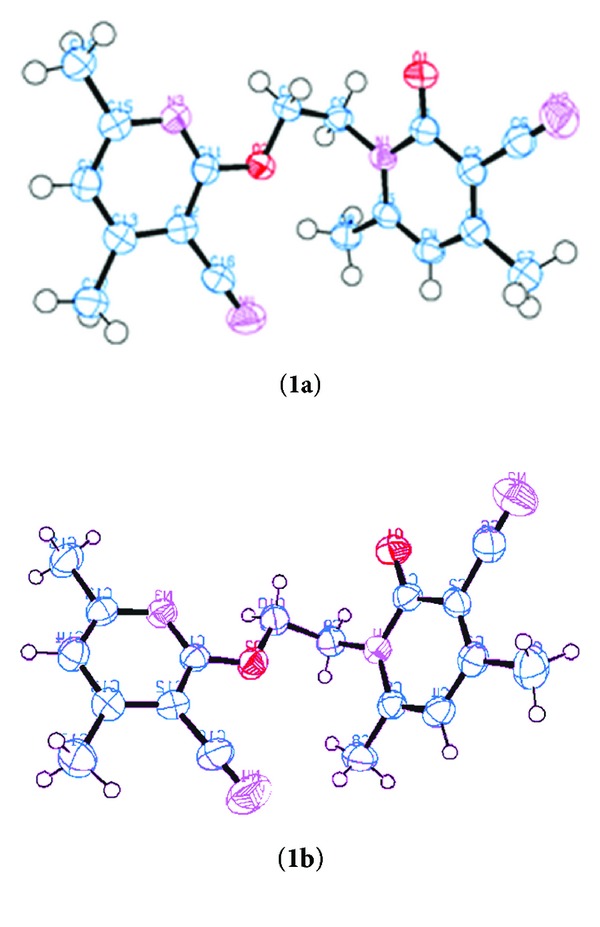
ORTEP diagram of polymorphs.

Crystal structure of the 1st polymorph (**1a**) and 2nd polymorph (**1b**) is shown in ORTEP diagram in Figure 1, respectively.

Weak aromatic interaction (CH⋯N, CH⋯*π*, and CH⋯O interaction) plays an important role in occupying both the polymorphs conformation. A detailed list of their bond lengths and bond angles are summarized in Table 2.

**Figure 2:**
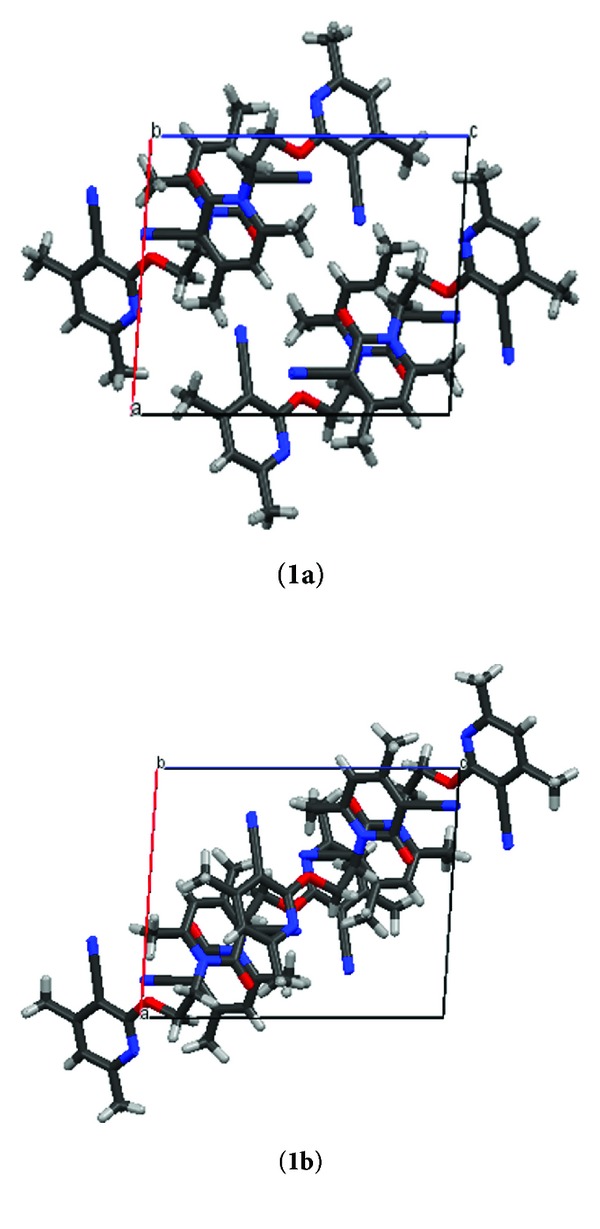
Packing diagram of 1st and 2nd polymorph along *b*-axis.

**Figure 3:**
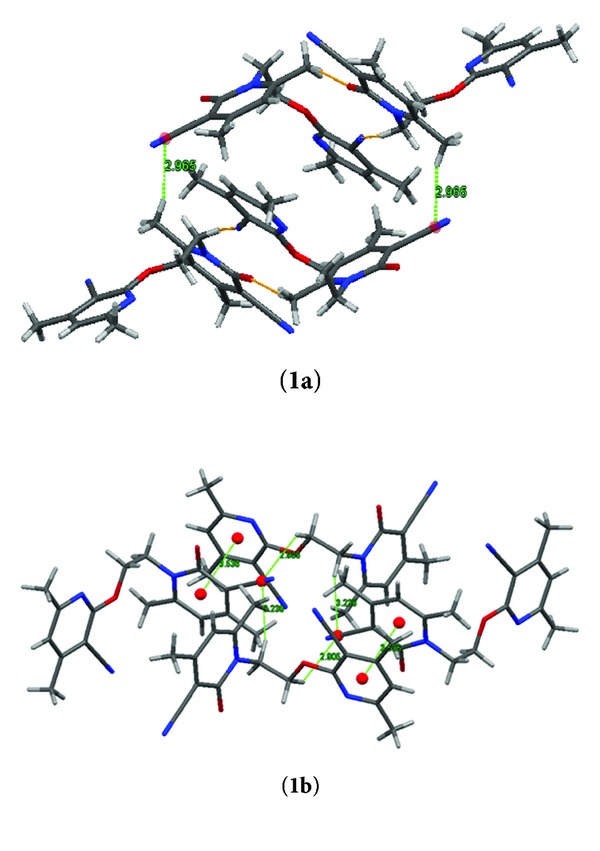
Packing of crystal shows its geometry and interactions in (**1a**) and (**1b**) polymorph.

 Intermolecular CH⋯N (2.573 Å, 131.53°) and CH⋯O (2.425 Å, 174.68°) interaction stabilized the network of **1a** in a symmetrical manner. However, these interactions are absent in polymorph **1b**. The major difference observed in the packing diagram of both the polymorphs (Figure 2) is that intermolecular *π*-*π* interaction present between centroid (C13C14C15N3C11C12) and centroid (C4C3C2C1N1C5) of heteroaromatic ring in **1b** is crystallized more closely while in the case of **1a** aromatic *π*-*π* interaction is completely absent and packing of this polymorph stabilized by CH⋯*π* interaction (Figure 3). 

Both polymorphs are showing roughness in their morphology due to the formation of zigzag sheets via weak interactions. In other words the crystal packing of molecules seems to achieve maximum crystal density. In the packing of the 1st polymorph **1a**, due to CH⋯O and CH⋯*π* (pi-bond of CN group) interaction, the molecules linked together and formed a cavity. However, in the case of **1b** the *π*⋯*π* and CH⋯*π* (pi-bond of CN group) interaction joined the molecules together in packing more tightly and a cavity appears. Presence of different sizes of cavities indicates that both the polymorphs can be used as a host for the different guest molecules. Such kinds of molecular systems will be helpful in many biological systems. Details of intermolecular weak interaction are given in Table 3.

*Docking Studies of Synthesized Compound.* Firstly, all bound waters, ligands, and cofactors were removed from the proteins. The macromolecule was checked for polar hydrogen; torsion bonds of the inhibitors were selected and defined. Gasteiger charges were computed and the AutoDock atom types were defined using AutoDock 4.2, graphical user interface of AutoDock supplied by MGL Tools [30]. The Lamarckian genetic algorithm (LGA), which is considered one of the best docking methods available in AutoDock [31, 32], was employed. This algorithm yields superior docking performance compared to simulated annealing or the simple genetic algorithm and the other search algorithms available in AutoDock 4.2. Secondly, the three-dimensional grid boxes were created by AutoGrid algorithm to evaluate the binding energies on the macromolecule coordinates. The grid maps representing the intact ligand in the actual docking target site were calculated with AutoGrid (part of the AutoDock package). Eventually cubic grids encompassed the binding site where the intact ligand was embedded. Finally, AutoDock was used to calculate the binding-free energy of a given inhibitor conformation in the macromolecular structure while the probable structure inaccuracies were ignored in the calculations. The search was extended over the whole receptor protein used as blind docking.

**Figure 4:**
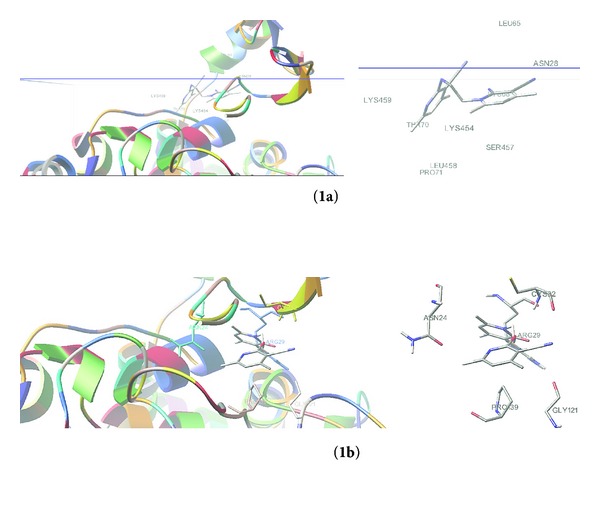
Docking analysis of both polymorphs.

The ability of compound **1a**-**b** to interact with the COX-2 was further assessed by *in silico *studies with AutoDock (Figure 4). Results indicate that polymorph **1b** shows a better binding effect with COX-2 compared with standard (Nimesulide) than **1a** (Table 4). It seems that **1b** can further be used as an anti-inflammatory drug. 

## 4. Conclusion

Weak interactions play an important role in stabilizing the structure of both polymorphs due to which they have different crystal packing. The presence of different sizes of cavities, formed via such weak interactions, plays a crucial role in their biological activity. Polymorph **1b** has more binding affinity with COX-2 than polymorph **1a**. Polymorph **1b** can further be explored for anti-inflammatory activity.

## Supplementary Material

CIF files of both the polymorphs are available.

## Figures and Tables

**Table 1:** Crystal data and structure refinement for polymorphs **1a** and **1b**.

Compound	**1a**	**1b**
Empirical formula	C_18_H_18_N_4_O_2_	C_18_H_18_N_4_O_2_
Formula weight	322.36	322.36
Wavelength	0.71073	0.71073
Crystal system	Monoclinic	Monoclinic
Space group	“P 21/n”	“P 21/n”
Unit cell dimensions (Å)	*a* = 10.025(2), *b* = 13.356(3), *c* = 11.964(2), *β* = 94.19(3)	*a* = 10.0026(4), *b* = 3.6580(8), *c* = 12.0838(7), *β* = 93.802(4)
Volume (Å^3^)	1597.6 (6)	1647.20 (15)
*Z*	4	5
Calculated density	1.340	1.484
Absorption coefficient	0.091	0.098
*F*(000)	680	780
*θ* range for data collection (°)	2.29–25.02	2.96–32.37
Limiting indices *h*, *k*, *l*	−11/11, −15/15, −14/14	−14/14, −18/20, −18/16
Refinement method	Full-matrix least-squares on *F* ^2^	Full-matrix least-squares on *F* ^2^
Final *R*1/*R*2 indices [*I* > 2 (*I*)]	*R*1 = 0.0505, *wR*2 = 0.1041	*R*1 = 0.0595, *wR*2 = 0.1660
*R* indices (all data)	*R*1 = 0.0528, *wR*2 = 0.1054	*R*1 = 0.2097, *wR*2 = 0.2105

**Table 2:** Intramolecular hydrogen: bonding geometry (Å and deg) for **1a **and **1b**.

D–H⋯A	**1a**				**1b**			
	*d*(D–H)	*d*(H⋯A)	*d*(D⋯A)	<(DHA)	*d*(D–H)	*d*(H⋯A)	*d*(D⋯A)	<(DHA)
CH_8C_⋯N4	0.980	2.915	3.496	118.96	0.959	2.664	3.544	152.32
CH_8C_⋯O2	0.980	2.489	3.237	132.93	—	—	—	—
CH_10A_⋯O1	0.990	2.660	3.145	110.36	—	—	—	—
CH_10B_⋯O1	—	—	—	—	0.970	2.671	3.144	110.38
CH_10B_⋯N3	0.990	2.564	2.713	87.82	0.970	2.770	2.700	75.75
CH_10A_⋯N3	0.990	2.799	2.713	74.77	0.970	2.554	2.700	88.00
CH_8C_⋯*π* (C16–N4)	0.980	2.912	3.607	128.73	0.959	2.851	3.654	141.84

**Table 3:** Intermolecular hydrogen: bonding geometry (Å and deg) for **1a** and **1b**.

D–H⋯A	**1a**				**1b**			
	*d*(D–H)	*d*(H⋯A)	*d*(D⋯A)	<(DHA)	*d*(D–H)	*d*(H⋯A)	*d*(D⋯A)	<(DHA)
CH⋯O	0.980	2.425	3.402	174.68	—	—	—	—
CH⋯N	0.990	2.573	3.313	131.53	—	—	—	—
CH⋯*π* (C*≡*N)	0.980	2.965	3.837	148.86	0.970	2.906	3.635	132.81
	—	—	—	—	0.970	3.238	4.135	154.55
*π*-*π*	—	—	—	—	—	3.536	—	—

**Table 4:** Compounds docking scores compared with indomethacin.

Compound no.	Docking score
**1a**	−7.36
**1b**	−7.63
Nimesulide	−7.59
